# Have rates of readmission for COPD been overestimated?

**DOI:** 10.1038/npjpcrm.2016.66

**Published:** 2016-10-13

**Authors:** Patrick T White, Timothy H Harries

**Affiliations:** 1 Department of Primary Care and Public Health Sciences, Division of Health and Social Care Research, King’s College London, London, UK

Hospital admissions and readmissions for chronic obstructive pulmonary disease (COPD) have been regarded as avoidable.^1^ In *npjPCRM* we reported on the stability of annual English national COPD admission data (2001–2010) presented as rates per 10,000 patients on a general practice list.^2^ Using these data we have derived national rates of COPD readmissions associated with these admissions. The derived COPD readmission rates are in keeping with other international studies.^3,4^ They stand in contrast to UK COPD readmission rates reported by the Royal College of Physicians (RCP) in 1997, 2003, 2008 and 2013. In this letter we compare the two approaches.

In their audits the RCP combined prospective identification of patients admitted with COPD with retrospective analysis of the outcome of that admission.^5–7^ Ninety days after admission, overall mortality was reported as 14.0% and readmission rate was 31–34%.^5,8^ The findings have led to a raft of recommendations encompassing all aspects of COPD care and including detailed discharge care bundles.^7^ We believe flaws in the method of the RCP Audit surveys make their findings unrepresentative of COPD admissions, and tend to undermine the evidence for their recommendations.

The RCP audits aimed to prospectively identify patients admitted with COPD. They specifically excluded patients in whom the diagnosis of COPD was applied after the initial assessment.^5^ This exclusion is likely to have skewed enrolment towards those patients at the most severe end of the COPD spectrum who would have been more easily identified on admission. Only 7% of patients in the latest RCP COPD audit were not known previously to have had COPD.^7^ There is considerable diagnostic uncertainty upon admission of a patient with worse breathlessness, sputum volume or sputum purulence to an Emergency Department or Medical Admissions Unit.^9,10^ This particularly affects the attribution of cause in a patient without a previous COPD diagnosis. A first admission is often the point of definitive diagnosis of COPD. The preferential inclusion of patients, the vast majority of whom were known previously to have had COPD, is suggestive of the probable greater severity of this group. They would have had an exceptional risk of readmission within 90 days and a low likelihood that they were representative of all COPD admissions. It is not specified whether only readmissions for COPD were included in the RCP audits, but the analysis and interpretation of all four audits imply that the readmission analysis was of COPD only.

The study we reported in the *npjPCRM* was based on COPD admissions reported by the NHS Information Centre Hospital Episodes Statistics database.^2^ Admission data were obtained for a sample of 806 English general practices that were representative of national practices in terms of COPD prevalence and deprivation score. The registered practice was recorded for every admitted patient. Every COPD admission was coded with the patient’s unique NHS number, enabling calculation of both the overall annual COPD admission rate and the annual rate of patients admitted with COPD per 10,000 patients on the GP list. These rates allowed calculation of the maximum annual COPD readmission rate per patient. Between 2001 and 2010, the mean annual number of patients admitted with COPD/10,000 patients ranged from 15.7 to 19.3, a variation of 22.9%. The mean annual number of COPD admissions/10,000 patients ranged from 24.3 to 28.3, a variation of 16.5%. Graphical representation of these rates with 95% confidence intervals demonstrated their relative stability.^2^ The denominator used, rates per 10,000 patients on a general practice list, enabled expression of the admission rate with relative confidence. More than 97% of the population in England is registered with a general practitioner with little duplicate registration.^11^

The *npjPCRM* paper shows that the annual difference between the rate of patients admitted with COPD and the overall rate of COPD admissions ranged between 2001 and 2010 from 6.1 (2008) to 10.1 (2003) COPD admissions/10,000 patients.^2^ This difference represents the excess of COPD admissions over the number of patients admitted with COPD. This excess is the rate of COPD readmissions within the year. On the basis of these data we have calculated readmission rates in this nationally representative sample. There were between 0.316 [6.1/19.3—the ratio in 2008] and 0.555 [10.1/18.2—the ratio in 2003] readmissions per patient per year, a range of one readmission per 8–14% of patients per quarter. This range is between a third and a half of the rate reported in the RCP audit.

The relatively low COPD readmission rates that we are reporting, allied to the long-term stability of COPD admissions, calls into question the validity of using COPD readmissions as a marker of quality of care. These findings together with our previous observations on the lack of evidence for an effect of primary care interventions on COPD admission risk emphasises the importance of directing efforts towards primary prevention of COPD and smoking cessation.^12^
